# Complete chloroplast genome sequence of the wild diploid potato relative, *Solanum acaule*

**DOI:** 10.1080/23802359.2021.1902414

**Published:** 2021-03-26

**Authors:** Tae-Ho Park

**Affiliations:** Department of Horticulture, Daegu University, Gyeongsan, South Korea

**Keywords:** Chloroplast, genome, genome sequence, *Solanum acaule*

## Abstract

*Solanum acaule* is a wild tuber-bearing species classified in the Solanaceae. The complete chloroplast genome of *S. acaule* was constructed by *de novo* assembly using Illumina paired-end (PE) sequencing data. The chloroplast genome of *S. acaule* is circular and has a length of 155,570 bp and typical quadripartite consisting of 86,020 bp of large single copy, 18,364 bp of small single copy, and 25,593 bp of a pair of inverted repeat regions. A total of 158 genes were annotated including 105 protein-coding genes, 45 *tRNA* genes, and eight *rRNA* genes. Maximum likelihood phylogenetic analysis of the sequence with 31 species in the *Solanaceae* revealed that *S. acaule* is fully resolved in a large clade with nine other *Solanum* species including *S. tuberosum*.

The wild tuber-bearing *Solanum acaule* is a potato relatives originating from Bolivia (Bitter 1912; Dvořák 1983; Hawkes 1990). Due to its resistance to various biotic and abiotic stresses, such as late blight, potato leaf roll virus (PLRV), potato virus Y (PVY), potato virus X (PVX), and frost, it is used as a source for potato (*S. tuberosum*) breeding (Chen et al. 1977; Estrada 1980; Chávez et al. 1988; Watanabe et al. 1994; Zoteeva et al. 2000). However, it is sexually incompatible and does not directly cross with *S. tuberosum*, even though both species are tetraploids. The endosperm balance numbers (EBNs) for these species are 2 and 4 in *S. acaule* and *S. tuberosum*, respectively (Hawkes 1990; Ortiz and Ehlenfeldt 1992; Cho et al. 1997). As a result, the wild species is difficult to use in potato breeding and more advanced methods, such as bridged cross and somatic hybridization have been attempted for breeding (Hermsen 1966; Hermsen and Ramanna 1973; Iwanaga et al. 1991; Yamada et al. 1998; Park et al. 2005; Rokka et al. 2005). Moreover, it is important to identify both the nuclear and cytoplasm genome composition after obtaining hybrids *via* bridged cross or somatic hybridization. Although molecular markers and genes, or cytogenetic analysis has been used to identify the nuclear genome contributions (Williams et al. 1990; Yamada et al. 1998; Spooner et al. 2008; Pendinen et al. 2012; Ono et al. 2016), the plastid genome of *Solanum* is rarely studied.

One of the lines of *S. acaule* (PI310970) was collected from Highland Agriculture Research Institute, South Korea (37°68′05.4′′N, 128°73′09.1′′E) and the specimen was deposited at the National Agrobiodiversity Center, South Korea (http://genebank.rda.go.kr/, Hyun-Jin Park, rosa2125@korea.kr) as the voucher number IT301483. Basically, the chloroplast genome sequencing was performed *via* Phyzen bioinformatics pipeline (Kim et al. 2015). Total genomic DNA was isolated using a Genomic DNA Extraction kit for plants (RBC, New Taipei City, Taiwan). The Illumina paired-end (PE) genomic library was constructed with the genomic DNA according to the PE standard protocol (Illumina, San Diego, CA). The library was sequenced using an Illumina HiSeq2000 platform at Macrogen (http://www.macrogen.com/kor/). In total, approximately 2.3 Gbp of raw data were produced and approximately 1.7 Gbp of high-quality of PE reads were obtained after trimming the sequences and removing low-quality bases with raw Phred score of 20 or less using the CLC quality trim program in the CLC assembly cell package version 4.2.1 (CLC Inc, Rarhus, Denmark). De novo assembly was performed with the trimmed reads obtained using the CLC de novo assemble program in the same package. The principal contigs representing the chloroplast genome were retrieved using Nucmer (Kurtz et al. 2004) with the chloroplast genome sequence of *S. berthaultii* (KY419708, Park 2017; Kim et al. 2018) as the reference sequence. The representative chloroplast contigs were arranged in order based on BLASTZ analysis (Schwartz et al. 2003) and connected to a single draft sequence by joining overlapping terminal sequences and manual editing through a comparison with the reference chloroplast genome sequence of *S. berthaultii* as described previously (Cho et al. 2015; Cho and Park 2016). The GeSeq program (Tillich et al. 2017) followed by manual curation using the results of BLAST searches were used for the annotation.

The complete chloroplast genome of *S. acaule* (GenBank accession no. MK036506) is 155,570 bp in length including two 25,593 bp inverted repeat (IRa and IRb) regions separated by small single copy (SSC) region of 18,364 bp and large single copy (LSC) region of 86,020 bp. When the chloroplast genome sequence was compared with those of the closest other *Solanum* species, such as *S. juzepczukii* (NC050208) and *S. megistacrolobum* (MH021517, MH021518, and MH021519), the sequence conservation between them was expectedly very high with a sequence identity of 99.90% and 99.88%, respectively. It has the typical quadripartite structure like most plastids and the structure and gene features were typically identical to those of higher plants. A total of 158 genes with an average size of 583.1 bp were annotated including 105 protein-coding genes, 45 *tRNA* genes, and eight *rRNA* genes with an average size of 764.6, 62.3, and 1,130.8 bp, respectively. An overall GC content was 37.22%.

The results from the phylogenetic analysis performed using chloroplast coding sequences of *S. acaule* and 31 published species belonging to the Solanaceae family by a maximum likelihood method with Kimura 2-parameter model and 1000 Bootstrapping options in MEGA version 6.0 (Tamura et al. 2013) revealed that *S. acaule* belonged to the same clade in tuber-bearing *Solanum* species as expected (Figure 1). Cytoplasmic genome types in *Solanum* species have been known to induce higher percentage of tuberization (Sanetomo and Gebhardt 2015) and plastomes of *Solanum* accessions in the section *Petota* were previously found to be highly similar, indicating a high degree of conservation (Huang et al. 2019).

**Figure 1.**
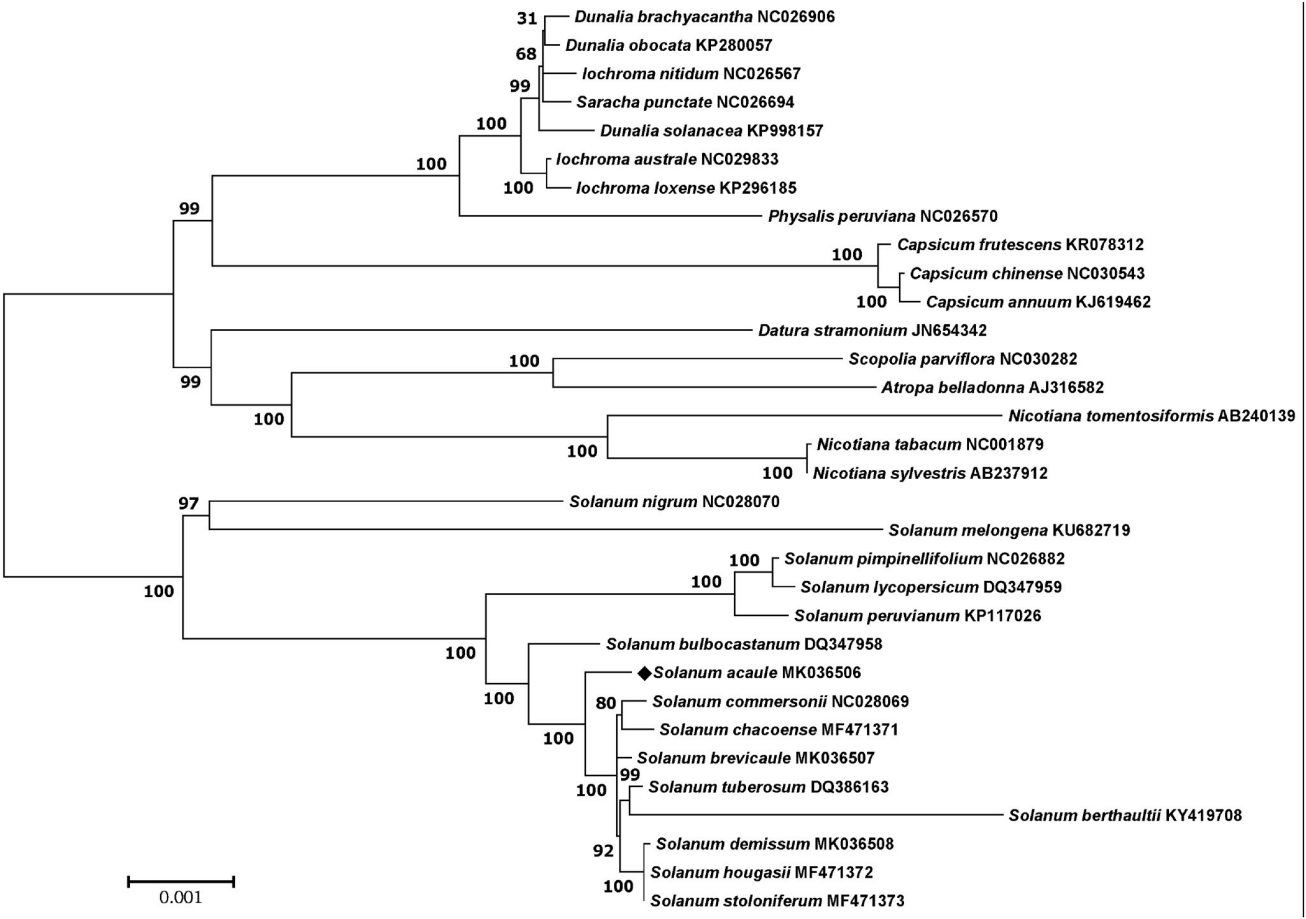
Maximum-likelihood phylogenetic tree of *S. acaule* with 31 species belonging to the Solanaceae based on chloroplast protein-coding sequences. Numbers in the nodes are the bootstrap values from 1000 replicates.

## Data Availability

The data that support the findings of this study are openly available in the NCBI under accession number MK036506 (https://www.ncbi.nlm.nih.gov/nuccore/MK036506). The associated BioProject, SRA, and BioSample numbers are PRJNA703768 (https://www.ncbi.nlm.nih.gov/bioproject/PRJNA703768), SRR13759748 (https://www.ncbi.nlm.nih.gov/sra/SRR13759748), and SAMN18024011 (https://www.ncbi.nlm.nih.gov/biosample/SAMN18024011), respectively.
